# Comparative genome-wide polymorphic microsatellite markers in Antarctic penguins through next generation sequencing

**DOI:** 10.1590/1678-4685-GMB-2016-0224

**Published:** 2017

**Authors:** Juliana A. Vianna, Daly Noll, Isidora Mura-Jornet, Paulina Valenzuela-Guerra, Daniel González-Acuña, Cristell Navarro, David E. Loyola, Gisele P. M. Dantas

**Affiliations:** 1 Departamento de Ecosistemas y Medio Ambiente, Pontificia Universidad Católica de Chile, Santiago, Chile; 2 Centro de Cambio Global UC, Santiago, Chile; 3 Departamento de Ciencias Pecuarias, Facultad de Ciencias Veterinarias, Universidad de Concepción, Chillán, Chile; 4 Centro Nacional de Genómica y Bioinformática, Santiago, Chile; 5 Pontifícia Universidade Católica de Minas Gerais, Belo Horizonte, MG, Brazil

**Keywords:** microsatellite markers, penguin, genome, polymorphism, tetranucleotide

## Abstract

Microsatellites are valuable molecular markers for evolutionary and ecological studies. Next generation sequencing is responsible for the increasing number of microsatellites for non-model species. Penguins of the *Pygoscelis* genus are comprised of three species: Adélie (*P. adeliae*), Chinstrap (*P. antarcticus*) and Gentoo penguin (*P. papua*), all distributed around Antarctica and the sub-Antarctic. The species have been affected differently by climate change, and the use of microsatellite markers will be crucial to monitor population dynamics. We characterized a large set of genome-wide microsatellites and evaluated polymorphisms in all three species. SOLiD reads were generated from the libraries of each species, identifying a large amount of microsatellite loci: 33,677, 35,265 and 42,057 for *P. adeliae, P. antarcticus* and *P. papua*, respectively. A large number of dinucleotide (66,139), trinucleotide (29,490) and tetranucleotide (11,849) microsatellites are described. Microsatellite abundance, diversity and orthology were characterized in penguin genomes. We evaluated polymorphisms in 170 tetranucleotide loci, obtaining 34 polymorphic loci in at least one species and 15 polymorphic loci in all three species, which allow to perform comparative studies. Polymorphic markers presented here enable a number of ecological, population, individual identification, parentage and evolutionary studies of *Pygoscelis*, with potential use in other penguin species.

## Introduction

The high level of polymorphism in microsatellites, or short tandem repeats (STRs), coupled with their codominant nature, is advantageous for population genetics or behavioral studies, such as paternity tests, investigation of mating patterns, phylogeography, etc. However, the identification of microsatellites in non-model species by traditional methods is a time-consuming process requiring extensive laboratory procedures and Sanger sequencing to achieve isolation of only a few loci per surveyed genome. The restricted number of loci identified through traditional methods may limit the results of a study. Therefore, most investigations on non-model organisms rely on cross-amplification of microsatellite loci from closely related species, a procedure whose applicability decreases as the evolutionary divergence among taxa increases. This is the case of bird species, for which microsatellite loci are much less abundant than for other vertebrate classes (Primmer *et al.*, 1997; Neff and Gross, 2001). Dawson *et al.* (2010) evaluated microsatellites across several bird species belonging to 15 orders and selected cross-species markers, thus reducing the costs and time associated with developing new ones. However, the use of microsatellites developed for different species is subject to scoring errors, such as null alleles, which can lead to biases in ecological and evolutionary conclusions drawn from the data (Dewoody *et al.*, 2006). Null alleles occur when one allele does not amplify because of mutations in the sequence where a primer was designed to anneal, which can often occur with unspecific primers (Dakin and Avise, 2004). On the other hand, using different microsatellites developed *de novo* for each species limits prospects of comparative studies between taxa. For that reason, identification of microsatellite loci common for a few closely related species of interest, allows comparative ecological and evolutionary inquiries.

Recent Next Generation Sequencing (NGS) technologies make it feasible to obtain a large number of markers (*e.g.* microsatellites, SNPs) and have thus revolutionized molecular studies in non-model organisms, permitting rapid characterization of gene structure and expression (Ellegren, 2008). Studies using neutral microsatellites can provide information to understand aspects of species’ ecology and population genetic structure (Freer *et al.*, 2015; Vianna *et al.*, 2017). Moreover, behavioral differences between males and females are frequently interpreted comparing population genetic patterns obtained from different markers such as mtDNA (maternal lineage) and microsatellite loci (biparental lineage; Freer *et al.*, 2015; Vianna *et al.*, 2017). An increasing number of genomes have recently become available, including those of several bird species (Jarvis *et al.*, 2014; Zhang *et al.*, 2014), with two species of penguins among them (Li *et al.*, 2014). Those studies focus on genome description and structure, phylogeny, adaptation, and comparative analyses. Although a large number of microsatellite loci are becoming available for new genomes, they are seldom evaluated with regard to their level of polymorphism in related species.

Antarctic organisms have been strongly affected by climate change during the past 50 years, mostly in the West Antarctic Peninsula (WAP), where effects are more drastic (*e.g.* Croxall *et al.*, 2002; Meredith and King, 2005; Montes-Hugo *et al.*, 2009; Trathan *et al.*, 2011). Genomic studies using NGS technology can, thus, provide different molecular markers to help understand adaptation, population dynamics and behavior of Antarctic taxa.

Penguins represent a monophyletic group from the Spheniscidae family, encompassing 18 species distributed exclusively in the Southern Hemisphere (Stonehouse, 1975; Williams, 1995). *Pygoscelis* penguins are comprised of three species, the Adélie penguin (*P. adeliae*), the Chinstrap penguin (*P. antarcticus*) and the Gentoo penguin (*P. papua*), all adapted to the cold temperatures around Antarctica and the sub-Antarctic islands. Among the three living species, *P. papua* has the most northern distribution along the Antarctic Peninsula and sub-Antarctic islands. *P. antarcticus* has a more southern distribution almost exclusively around the Antarctic Peninsula, and *P. adeliae* is the most dependent on ice, reaching higher latitudes and a circumpolar distribution (Stonehouse, 1975). *P. adeliae, P. papua* and *P. antarcticus* are classified by the IUCN as Least Concern (IUCN - International Union for Conservation of Nature, 2017).

Molecular DNA studies of *Pygoscelis* were mostly restricted to sequences (Ritchie and Lambert, 2000; Ritchie *et al.*, 2004; Peña *et al.*, 2014; Clucas *et al.*, 2014), except for a study of *P. adeliae* using six dinucleotide microsatellites (Roeder *et al.*, 2001). These were developed by traditional methods using genomic libraries (Roeder *et al.*, 2001; 2002) and then investigated for cross-amplification in 16 other species of penguins, most of which were monomorphic (Roeder *et al.*, 2002). Recently, Kang *et al.* (2015) isolated sixteen polymorphic microsatellite loci for the chinstrap penguin (*P. antarcticus*), while Li *et al.* (2014) sequenced the genomes of *P. adeliae* and *Aptenodytes forsteri* to study the phylogenetic and population history of penguins, but neither evaluated microsatellites for polymorphisms.

The three *Pygoscelis* species in this study have suffered the effects of climate change, with reductions in population size, changes in distribution, and even local extinction. The WAP, a large area where these species are found, is the Antarctic region that has been most affected by climate change. Therefore, genetic studies of population structure, using variable molecular markers, are important in monitoring these Antarctic species in space and time.

We sequenced the genomes of *Pygoscelis adeliae, P. antarcticus* and *P. papua* using the Applied Biosystems Support Oligonucleotide Ligation Detection (SOLiD) platform, and identified and characterized microsatellite loci evaluating and comparing structure (motif class and type) among species. We also report a genome-wide set of several microsatellite loci for cross amplification in all three *Pygoscelis* species and the evaluation of the degree of polymorphism of several tetranucleotide loci. Finally, we report a set of primers designed for several microsatellite loci. These have similar melting temperatures and allow multiplex amplification using the same PCR protocol. These loci can be an important resource for future genetic studies of penguin populations to help management and conservation of these species in the face of climatic change.

## Material and Methods

### SOLiD sequencing

Genomic DNA was isolated from *Pygoscelis papua, P. antarcticus* and *P. adeliae* blood samples preserved in ethanol using the salt method (Aljanabi and Martinez 1997). DNA from six individuals belonging to the three species was quantified and quality checked by fluorometry using the PicoGreen®assay kit (Invitrogen). Genomic sequencing in ABISOLiD 5500 XL was performed at Omics Solution, a Next Generation Sequencing facility (Santiago, Chile). DNA was desalted and then concentrated using standard EtOH/sodium acetate precipitation at 20 °C for 2 h, followed by two 70% EtOH washes. The pooled DNA was re-dissolved in low TE as per standard protocol for ABI SOLiD sequencing of genomic DNA fragment libraries. DNA samples were sheared in a CovarisS220 System (Thermo Fisher Scientific), which sonicates the input DNA into small fragments with a mean size of around 160 bp.

The fragmented DNA was then purified with SOLiD Library Column Purification Kit (Thermo Fisher Scientific), and libraries were prepared according to standard SOLiD protocols. Fragment libraries for the twelve penguin samples were prepared separately as follows: P1 and P2 adapters (Thermo Fisher Scientific) were ligated, and each sample was tagged with a different barcode (a known adapters sequence of ten bp). Prepared libraries were quantified by real-time PCR in a Light Cycler®Nano (Roche) using the Quantification Kit for SOLiD (Invitrogen). Each double stranded library was added at a concentration of 0.2 pg/μL to the emulsion with 2,400 million beads, according to the manufacturers’ instructions. Thirty percent of the beads were P2 positive (contained amplified library fragments) before enrichment, and 90% of the beads were P2 positive after enrichment, yielding 790 million beads deposited in the Flow Chip. Library beads were sequenced in a SOLiD 5500 XL using standard chemistry for paired-end fragment libraries and 35-75 bp read lengths.

### SOLiD sequence alignment

The color-space reads (di-base encoded) were aligned with LifeScope software (Applied Biosystems) using the genome assembly of *P. adeliae* (Li *et al.*, 2014) as reference. The reference was translated into color-space with the aim of mapping the reads. The color-space reads helped to improve the quality of each base call, since each base was read twice during the sequencing step.

The consensus sequence was built from the binary alignment map (BAM) files obtained in the previous step. We used the SAMtools (Li e*t al.,* 2009) repositories to obtain all bases mapped to each position, BCF tools to get the most probable genotype per position, and VCF utilities to build the consensus sequence in FASTQ format. The FASTQ file was then converted to FASTA using SEQTK (Li, 2012).

### Identification of microsatellite loci

The search for dinucleotide, trinucleotide, and tetranucleotide tandem repeats in the obtained scaffolds were done using MISA software (http://pgrc.ipk-gatersleben.de/misa/). We designed primers to amplify repeat fragments of 200 bp or less. Oligonucleotide primer pairs flanking the microsatellite sequence were designed using Primer 3.0 software (Rozen and Skaletsky, 2000; http://primer3.sourceforge.net/) based on the following parameters: product size of 150-250 and 250-300 bp; TM of 60 °C, ranging between 58 and 63 °C. Reverse-complement repeat motifs (*e.g.* TG and CA) and translated or shifted motifs (*e.g.* TGG and GTG) were grouped together such that there were a total of four, 10 and 33 unique dinucleotide, trinucleotide and tetranucleotide repeats, respectively.

### Microsatellite isolation and evaluation

From our list of potential primers present in *P. papua* (Gentoo penguin, GP), *P. antarticus* (Chinstrap penguin, CP) and *P. adeliae* (Adélie penguin, AP) (Table S1), we chose a subset for evaluation in all three species. The first criterion for selection was a tetranucleotide, simple and perfect SSR repeat motif and TM of 60 °C. Primer pairs for each locus were evaluated using the AmplifX v. 1.5.4 software (http://crn2m.univ-mrs.fr/pub/amplifx-dist) considering the stability of the PCR reaction, percentage of GC, stability at 3′, the absence of dimers or hairpins. Although tetranucleotide microsatellites may have lower mutation rates than dinucleotides (Kruglyak *et al.*, 1998; Schug *et al.*, 1998), we decided to carry on our analysis using polymorphic tetranucleotides that amplify in all three penguin species. Amplification of artifact bands (or stutter bands) in dinucleotide repeats can lead to misidentification of an allele, something that is less likely to happen in tetranucleotide repeats. Tetranucleotide microsatellites have shown higher discriminatory power among closely related populations than most dinucleotides (*e.g.*, Greig *et al.*, 2003; Huang *et al.*, 2015). Hence, tetranucleotides are becoming increasingly popular markers because allele differences are easier to distinguish than those of dinucleotide repeats.

Therefore, 170 loci of tetranucleotide SSR repeat motifs were selected to evaluate polymorphisms in each *Pygoscelis* species. PCR reactions incorporated the forward primers with 5′-end-M13 tail, the fluorophorelabeled M13 primer (Schuelke, 2000) with 6-FAM, NED or HEX (Applied Biosystems) and a reverse primer. The analysis was done on 26 samples, nine from *P. papua*, nine from *P. antarcticus* and eight from *P. adeliae*, with individuals from three, three and two different sampling locations, respectively.

We searched for orthology for all tetranucleotide loci between pairwise species and all three species considering 100% identity between the primers sequences using the option Find Duplicates in Excel and the criteria of location at the same scaffold and same locus motif.

Polymerase chain reactions (PCRs) were carried out in a 30 μL volume containing 2 μL of DNA at 25 ng/μL, 1X reaction buffer, 1.5 mM of MgCl_2_, 200 uM of each dNTP, 0.4 μM of each primer, and 0.8 units of *Taq* DNA polymerase (Brasil, Invitrogen). The PCR protocol was as follows: 10 min at 95 °C, a touchdown series of 95 °C for 15 s, 60–50 °C for 30 s, 72 °C for 45 s, with two cycles at each annealing temperature, and 35 amplification cycles of 95 °C for 15 s, 50 °C for 30 s, 72 °C for 45 s, followed by a final extension step of 30 min at 72 °C. All PCR products were loaded on 3% agarose gels with SB buffer (Brody and Kern, 2004), and also on non-denaturing 12% acrylamide gels (99:1 acrylamide: bis-acrylamide), and run for 0.5 h at 300 V, and 3 h at 300 V, respectively. Bands in agarose gels were visualized with GelRed on an UV transilluminator, and acrylamide gels were stained with silver nitrate.

For our purposes, PCR amplification of a microsatellite was considered successful if a band of the expected size was observed after gel electrophoresis, even if two or more bands were amplified. Monomorphic tetranucleotide loci observed were discarded, and all loci with more than one allele were genotyped at Macrogen Inc. (Korea). Electrophoretograms were analyzed using GeneMarkerv1.75 (Softgenetics LLCTM, State College). A microsatellite locus was considered successful if the resulting electrophoretograms showed at least two alleles per locus in all three species, but never more than two alleles in a single individual. The reads containing polymorphic tetranucleotides were deposited in GenBank under accession numbers KU182396–KU182429.

The number of observed alleles (NA) and observed heterozygosities (*Ho*) were calculated using Arlequin 3.5 (Excoffier and Lischer, 2010). The probability of identity (PI) and PI between siblings (PIsibs) was calculated using GenAlEx 6.5 (Peakall and Smouse, 2012). PI values estimate the probability that two individuals, taken at random from a population, will have the same genotype at multiple loci (Waits *et al.*, 2001). PI is calculated to determine the number of loci required to resolve individual identity within populations by gradually increasing the number of loci, adding the most variable loci first (Waits *et al.*, 2001). PIsibs is a similar but more conservative estimate of the number of loci required to establish individual identity.

Fifteen polymorphic loci were then evaluated for one population of *P. papua* (n = 30) and one of *P. antarcticus* (n = 30). These loci were selected based on the criteria of being polymorphic for all three *Pygoscelis* species (11 of 15 loci selected) or polymorphic for one or both species (4 loci). The number of observed alleles (*N*_*A*_) and observed (*H*_*o*_) and expected heterozygosities (*H*_*e*_) were calculated using Arlequin 3.5 (Excoffier and Lischer, 2010). Deviations from Hardy-Weinberg equilibrium were calculated with the *F*_*IS*_ index in Genetix v. 4.05 (Belkhir *et al.*, 2004).

## Results

For the *Pygoscelis* genome, a total of 59,889,052 filtered reads and 52,997,551 aligned reads were obtained for *P. adeliae*, 186,606,554 filtered reads and 158,565,602 aligned reads for *P. papua* and 94,746,776 filtered and 80,496,366 aligned reads for *P. antarcticus* (Table 1).

**Table 1:** Number of reads (filtered and aligned), number of microsatellite loci and motifs for all three *Pygoscelis* species, including the total of loci and only those that showed flanking region sequence for primer design.

	*P. papua*	*P. antarcticus*	*P. adeliae*
Filtered reads	186,606,554	94,746,776	59,889,052
Aligned reads	158,565,602	80,496,366	52,997,551
No of loci	42,057	35,265	33,677
No of loci with primers	29,618	17,777	13,492
Total of Dinucleotide	24,528	21,250	20,361
Total of Trinucleotide	11,667	9216	8,607
Total of Tetranucleotide	4,370	3,788	3,691
Dinucleotide with primers	18,639	11,449	8,673
Trinucleotide with primers	7,385	4,314	3,198
Tetranucleotide with primers	2,699	1,594	1,287

### Identification of microsatellite markers

We identified a total of 35,265, 42,057, and 33,677 microsatellite loci in the genomes of *P. antarcticus, P. papua*, and *P. adeliae*, respectively. Among these markers were 17,777, 29,618, and 13,492, respectively, which showed flanking region sequences suitable for primer design. We identified a total of 38,761 dinucleotide, 14,897 trinucleotide and 5,580 tetranucleotide repeats with the primer design in all three genomes (Table 1). The microsatellite loci turned out to be genome-wide distributed across different scaffold (Table S1). Loci motifs, primer sequences, melting temperatures, product size and scaffold for 5,580 tetranucleotides that occur in all three *Pygoscelis* genomes are included in Table S1 as well. From these 5,580 tetranucleotide loci, 70 were confirmed to be orthologous for all three species, showing 100% identity for both primers sequences (forward and reverse) for the same locus and showing the same scaffold and same locus motif (Table S1). However, this number may be underestimated since the comparison considered 100% identity for both primers designed independently between species. In Pairwise species comparisons we found orthology in 477, 245 and 142 loci between GP/CP, GP/AP, AP/CP respectively.

**Figure 1:**
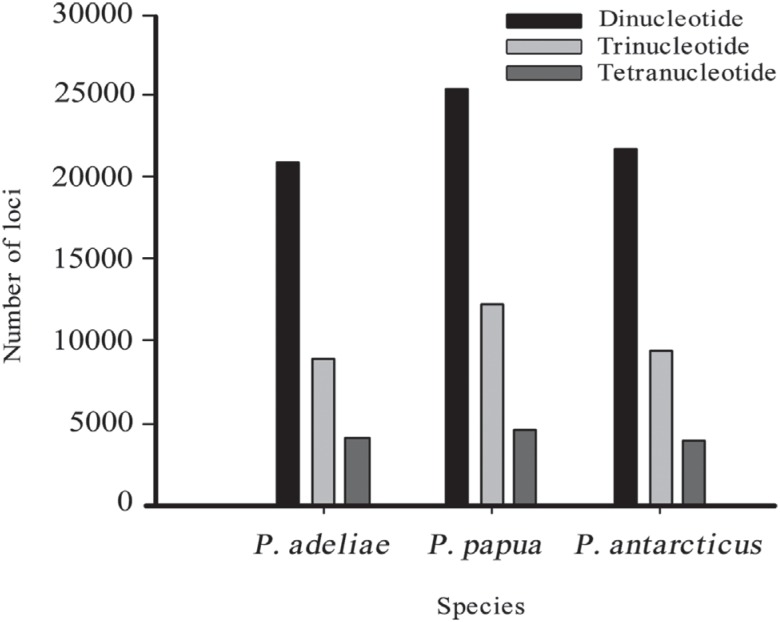
Numbers of identified dinucleotide (black), trinucleotide (light gray) and tetranucleotide (dark gray) microsatellite repeat loci, for *P. adeliae, P. papua and P. antarcticus* genome.

**Figure 2:**
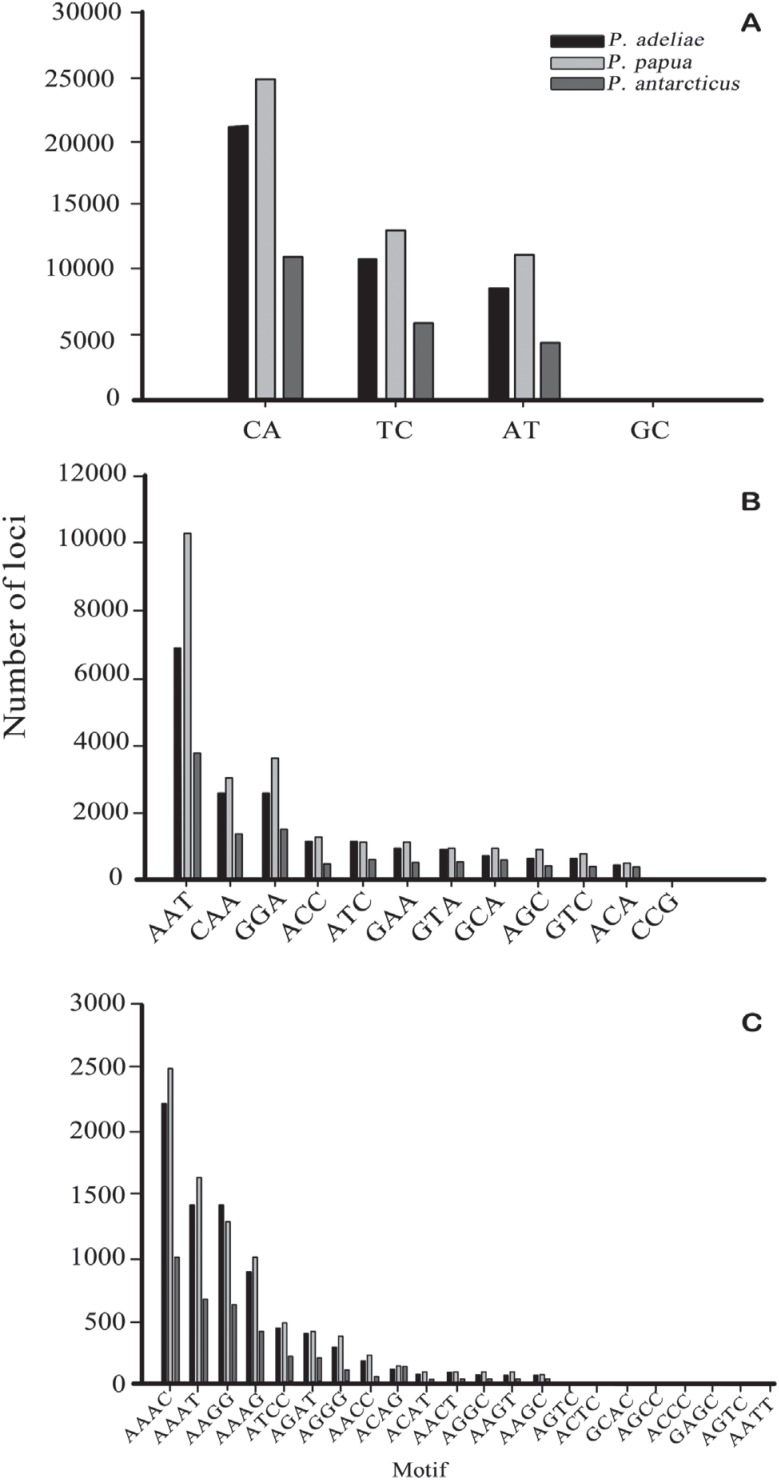
Number of dinucleotide (A), trinucleotide (B) and tetranucleotide (C) loci with different motifs identified in *P. Adeliae* (black bar), *P. papua* (light gray bar) and *P. antarcticus* (dark gray bar).

All species showed higher and similar numbers of dinucleotides, followed by trinucleotides and tetranucleotides (Figure 1). The frequencies of specific repeat motifs were similar for the three species. For dinucleotides, CA repeats were the most frequent across all species, followed by TC, AT and a reduced number of GC loci (Figure 2A). For trinucleotides, AAT was the most abundant, followed by CCA and GGA (Figure 2B). For tetranucleotides, the most frequent repeat was AAAC, followed by AAAT and AAGG (Figure 2C).

### Polymorphism of microsatellite loci for *Pygoscelis* species

From the 60,877 primers designed, 170 were selected to evaluate polymorphisms in *Pygoscelis*: 90 loci based on the *P. adeliae* genome, 40 on *P. papua* and 40 on *P. antarcticus*. Of these, 34 were easily amplified in all three species using the same PCR conditions and were visibly polymorphic at electrophoretic mobility. These 34 loci were also genome wide distributed, being mostly identified in different scaffolds, with exception of six loci which were found in three different scaffolds (Table 2). Among these 34 loci, 14 were isolated from *P. adeliae* genome, 11 from *P. papua*, and 9 from *P. antarcticus*. We evaluated these loci in 26 individuals, where 15 loci were polymorphic in all three species, 13 were polymorphic in two species and 6 were polymorphic in one of the three species (Table 2). Therefore, a total of 30 polymorphic microsatellites were identified in *P. adeliae*, 25 in *P. papua*, and 22 in *P. antarcticus*. Out of all 15 polymorphic loci, the number of alleles per species and per locus varied between 2 and 8. The highest number of alleles per locus was found in *P. adeliae* (mean 4.87; SD 1.36), followed by *P. antarcticus* (mean 4.47; SD 1.68) and *P. papua* (mean 3.53; SD 1.25). Heterozygosity was always higher for *P. adeliae* (*H*_*o*_ = 0.59), followed by *P. papua* (*H*_*o*_ = 0.54) and *P. antarcticus* (*H*_*o*_ = 0.42) in all 15 loci. Moreover, when we compared the statistics between microsatellite markers isolated from each species’ own genome with those isolated from other species’ genomes, we found similar ranges of allele numbers and heterozygosity (Table 3). This suggests that these markers are not subject to decreased heterozygosity among species of this genus due to cross-species amplification. These results receive support from the orthology of those markers for all three species. Fifty two percent of the microsatellite loci from those 34 selected according to their polymorphism were identified in two or all three *Pygoscelis* species and 100% were identified at the same scaffold, and frequently showed the same primer sequence between species. These orthologous percentages were significantly reduced to 3 of 34 loci when we considered 100% identity for both primers simultaneously.

**Table 2:** Tetranucleotide microsatellite loci polymorphic in all three *Pygoscelis* species (P3), in two species (P2), and one (P1), with their respective scaffold, locus, primer sequence, motif, number of samples (N), number of observed alleles per marker (A) and observed heterozygosity (*H*_*o*_).

					*Pygoscelis adeliae*				*Pygoscelis papua*				*Pygoscelis antarcticus*			
	Scaffold	Locus	Primer sequence (5′-3′)	Motif	Size (bp)	N	A	Ho	Size (bp)	N	A	Ho	Size (bp)	N	A	Ho
P3	51	AP-26	F: TGGGGAGCAGAGTATTTTTGTT	(GATA)7	205-245	6	8	0.67	217-241	9	6	0.78	231-263	9	5	0.56
			R: AGCCACGAAAAGTGAGCCTA													
	46	AP-61	F: GCTCTCAGTTGAGCAAACCC	(TTTG)6	230-242	8	4	0.63	216-228	9	2	0.67	230-250	9	6	0.78
			R: TTCGTGCTTCTTGCTTTCCT													
	181	AP-78	F: CCACTGCAGTATCCCATTTTT	(TATC)6	188-200	8	4	0.50	188-208	9	5	0.56	188-208	9	3	0.22
			R: GGTCAAAGCTTACCATCCCA													
	27	AP-85	F: CAAAACAGTCACTAGTGTGCCA	(ATCT)9	212-232	8	6	1.00	188-204	9	4	0.44	204-210	9	4	0.44
			R: TACGCCAATGAAAAGCACTG													
	44	AP-90	F: TGCATGTTGGAACATCAAAA	(GATA)7	162-178	8	5	0.88	194-206	9	3	0.78	162-186	9	7	0.44
			R: AACACACACACGTGCACTCTT													
	18	GP-4	F: TCATCACCAATGGTTCAGAAA	(ATGA)5	212-232	8	6	1.00	256-264	9	3	0.44	268-284	9	5	0.78
			R: CCATGTGGTATTCATTCtGGTG													
	100	GP-6	F: AACATCTCATGAAGGCACAGC	(CATC)5	234-246	8	4	0.63	230-238	9	3	0.67	234-238	9	2	0.11
			R: GTACCATGCCCTTTCTGATGA													
	57	GP-13	F: CACATCCTTTCCCTCTCTTCC	(AGAT)11	182-202	7	4	0.29	194-202	9	3	0.67	190-202	6	3	0.33
			R: AAGGAGYGTGCCTAGTTTTGG													
	86	GP15	F: CTGTATTGAGATGGCCGTTGT	(CATC)7	250-274	8	7	0.38	254-266	9	5	0.44	250-274	9	4	0.22
			R: GTACCCACCGACTTCTCTTCC													
	101	GP-18	F: GGTCACCATGAGCAGTCAGTT	(ATAG)10	194-206	8	4	0.63	190-202	9	3	0.22	194-202	9	2	0.11
			R: TGAAAAGCTCTCCCCACAGTA													
	356	GP-19	F: TTGGGGAAATGACAACCCTAT	(TATC)5	223-239	7	4	0.71	227-235	9	3	0.67	221-227	9	3	0.22
			R: CCCTCACTGCTCAAGTCTGTC													
	241	GP-36	F: CTGTAAGTCACAGCGTGCAAA	(ATAC)5	248-256	8	3	0.25	248-268	8	5	0.50	252-264	6	4	0.17
			R: TGTGAGAACCATTGGACTTGT													
	240	CP-6	F: AGGCTTTCTCACACTGTGCTC	(CTAC)5	212-232	8	5	0.88	212-216	9	2	0.22	204-228	9	6	0.67
			R: AATGAGCAATTCAGGATGGTG													
	174	CP-25	F: GTCAAAGCCTGCRTCAACTCT	(CATC)13	196-224	7	4	0.43	200-216	9	5	0.78	208-242	8	7	0.75
			R: ATGACACTGGCAAAGGAGATG													
	189	CP-29	F: GCATCAGATCCCAGAATACCA	(AGAC)5	184-216	7	5	0.57	180-188	9	2	0.11	188-192	8	2	0.13
			R: TCTGGCAGTATGGGAAAACAC													
P2	108	AP-3	F: AAGCAGGGAAACCATACAAAGA	(ATAA)7	174-186	8	3	0.38	182-190	9	2	0.78	178	9	M	–
			R: GGTCTGAGTAAGGCTCTTCAGG													
	197	AP-12	F: AGGACAACGAGGCGAGAGT	(GAAG)5	212-216	8	2	0.25	212	9	M	–	212-216	9	2	0.33
			R: CCCTCCACCCTTTCTCTCTT													
	218	AP-14	F: TGGTAAACAGTCACACGGGA	(AAAC)5	224-228	8	2	0.75	228	9	M	–	220-224	9	2	0.22
			R: CCAAAACTGAGAAGCAACCC													
	37	AP-19	F: CCAGTGTTTGACCACCCTCT	(ATAA)7	250-258	8	3	0.5	246	9	M	–	250-262	7	4	0.71
			R: TGCATTTTTCCATTCCATTTC													
	590	AP-29	F: CACATCCGTGTGTTGGAAAG	(CATC)5	256-260	8	2	0.13	256-260	9	2	0.22	256	9	M	–
			R: GTTGGCGTTAACTGGGAAGA													
	18	AP-79	F: TTTTTCAAGGTAGAGGGCTCA	(TAGA)8	174-190	8	4	0.75	170-174	9	2	0.67	178	9	M	–
			R: GGAAGATAATATTTTGCATTTTCA													
	30	AP-87	F: GCCTTCCCATCTGTAGAAGC	(TATC)9	170-224	8	9	0.75	174	9	M	–	162-166	9	2	0.33
			R: TTTTCCAAAGTACGCAAGCA													
	9	GP-2	F: AGCACCACTCTCTCTTCCTCC	(AGTA)5	234-242	7	3	0.57	234-242	9	2	0.11	234	9	M	–
			R: GCAATTTCTTTTGAAACCCCt													
	349	GP-30	F: TTCCTCTACCYGCCTCAATTT	(CAGA)7	158-174	7	4	0.71	170-182	9	4	0.33	170	9	M	–
			R: TTACCTTTTCCTGTGCCCTTT													
	21	CP-5	F: CCAAATAGTCCCCCAAACCTA	(CATC)8	226-238	7	2	0.29	218	9	M	–	225-234	9	3	0.56
			R: GGGATAAAAATGGATGGATGAA													
	38	CP-17	F: TGAGTCATTTTGCAACTGGTG	(TAGA)5	255-267	8	4	0.75	255-263	9	3	0.67	259	9	M	–
			R: GAATGCACGCTGAAAAGAAAG													
	203	CP-22	F: AAAGAAGCTGGAGCCAAACT	(GATG)5	206-210	7	2	0.29	206-214	9	3	0.66	206	9	M	–
			R: ACCCCAGTGCTTCCATGTATT													
	175	CP-27	F: CACACTCCATATTGCCACACA	(TATG)5	170	8	M	–	158-170	9	2	0.11	170-174	9	2	0.44
			R: TTCAGAAAGTGGCTCCAAGA													
P1	367	AP-52	F: GCCTCAAACAGGACAGAAGC	(AAAC)5	224	8	M	–	216-228	9	3	0.67	224	9	M	–
			R: GGAATGGCTTCTGGTTGAAA													
	44	AP-57	F: CTGCTAGCCTTAGGCATTGG	(CAAA)6	230	8	M	–	228	9	M	–	222-234	9	4	0.44
			R: CTTTTGCCTTCTGCCTTGTC													
	92	GP-1	F: ATACCTGTGCTCATCTGTGGG	(AGCA)5	240-244	8	2	0.38	240	9	M	–	240	9	M	–
			R: AATGAGGTAGCACCCTGGACT													
	54	GP-24	F: ACGCATGAAGTAGGCAAGAGA	(GATA)10	233-237	8	2	0.25	233	9	M	–	233	9	M	–
			R: TACGAAGTGGTGGTGAAGAC													
	27	CP-4	F: GGAAACCAAAATCATCCATCC	(CATC)6	252	8	M	–	248-252	9	2	0.11	248	9	M	–
			R: AGGACCTGCATCTTTTCCAGT													
	7	CP-21	F: AGATGACAGTCTGGGGAAAGG	(ATAG)6	162-190	7	7	0.43	158	9	M	–	158	9	M	–
			R: CTCCCAAGGAAAACCTACCAG													

**Table 3:** Mean number of alleles per marker (*N*_*A*_) and observed heterozygosity (*H*_*o*_) for microsatellite loci isolated from all three species. (AP: *P. adeliae,* GP: *P. papua* and CP: *P. antarcticus*).

Microsatellite loci	*P. adeliae*		*P. papua*		*P. antarcticus*	
	N_A_	H_O_	N_A_	H_O_	N_A_	H_O_
AP	3.85	0.52	2.43	0.33	3.07	0.9
GP	3.91	0.53	2.91	0.40	2.82	0.25
CP	3.56	0.23	2.33	0.22	2.67	0.17

When evaluating 12 loci for one population of *P. papua* and *P. antarcticus* (n = 28-30), we observed that the number of alleles per species per locus varied between 1 to 7 and 1 to 10, respectively (Table 4). Only two loci showed departure from Hardy-Weinberg Equilibrium for *P. papua* and three for *P. antarcticus*. The probability of identity (PI, the probability of two independent samples having an identical genotype), based on all 15 microsatellites, resulted in values as low as 1.4E-13, 2.5E-9, and 1.2E-11, and PIsibs (probability of identity when random siblings are included in the samples) as low as 5.5E-06, 1.5E-04, and 2.8E-05 for *P. adeliae, P. papua* and *P. antarcticus*, respectively (Figure S1). PI was close to zero when combining only the three most polymorphic loci and the same effect was seen in PIsibs when combining the six most polymorphic loci. Markers with the lowest PI are GP-15 (4.8E-02), AP-26 (5.2E-02), and GP-04 (6.7E-02) in *P. adeliae*; AP-26 (1.3E-01), CP-25 (1.3E-01), and GP-36 (1.3E-01) in *P. papua*; and CP-25 (6.9E-02), GP-6 (7.6E-02), and AP-90 (8.4E-02) in *P. antarcticus*.

**Table 4:** Tetranucleotide microsatellite loci polymorphism for 30 individuals and 12 loci for *P. papua* and *P. antarcticus*, (N: Number of observed alleles per marker; *H*_*o*:_ Observed heterozygosity, *H*_*e*:_ Expected heterozygosity, *F*_*IS*_ value).

	*Pygoscelis papua*						*Pygoscelis antarcticus*					
Locus	Size (bp)	N	A	Ho	He	*F*_IS_	Size (bp)	N	A	Ho	He	*F*_IS_
AP-3	178-186	30	2	0.37	0.49	0.261*	174-178	30	2	0.067	0.065	-0.018
AP-19	246	30	1	0	0		250-290	28	8	0.82	0.79	-0.034
AP-26	225-241	30	6	0.70	0.77	0.100	231-255	30	6	0.8	0.8	0
AP-61							218-250	28	8	0.643	0.755	0.151*
AP-78	188-212	30	7	0.80	0.69	-0.169	188-208	30	4	0.266	0.38	0.309*
AP-85	188-208	30	5	0.67	0.70	0.051	200-224	30	7	0.700	0.763	0.084
AP-90	194-214	30	6	0.53	0.56	0.045	158-190	30	9	0.900	0.840	-0.072
GP-6	230-234	29	2	0.28	0.29	0.051	234	28	1	0	0	
GP-13	190-206	30	5	0.90	0.62	-0.479						
GP15							250-286	30	10	0.767	0.806	0.049
GP-36	240-260	30	5	0.13	0.16	0.171*	244-260	30	5	0.333	0.473	0.30*
CP-6							228-236	30	3	0.600	0.524	-0.146
CP-17	255-259	29	2	0.10	0.09	-0.037						
CP-22	190-234	30	7	0.73	0.76	0.311						
CP-25	192-212	30	6	0.63	0.55	-0.152	204-268	30	10	0.866	0.826	-0.05

* Significant *F*_*IS*_ values, p < 0.05.

All microsatellite loci were amplified under the same PCR condition, which means they can be used in multiplex PCR assays. A multiplex microsatellite genotyping run can genotype a total of six loci for each species using three different stains, or eight loci using four stains (*e.g.* VIC, NED, 6-FAM, PET, HEX, depending on the equipment). We can select loci to be used in all three species according to size range: fragments smaller than 214 bp (AP-78, AP-90, GP-13, GP-18) can be combined with fragments greater than 221 bp (GP-6, GP-15, GP-19, GP-36). The remaining loci can be selected for each species individually. Taking *P. papua* as an example, it is possible to combine loci greater than 212 bp in length (CP-06, AP-61, AP-26 and GP-4) with some under 204 bp (AP-85, CP-29).

## Discussion

### Comparative analysis of microsatellites in the three genomes

The SOLiD™ next-generation sequencing (NGS) platform allowed identification of 110,999 microsatellite loci (di-, tri-, tetranucleotide), with an average of 36,999 loci per *Pygoscelis* species. Most studies rely on different NGS platforms for screening small parts of the genome for microsatellites. Illumina and 454 platforms have dominated the identification of microsatellite markers in non-model organisms (Moodley *et al.*, 2015, Zalapa *et al.*, 2012). Other platforms, such as Ion Torrent PGM and Illumina MiSeq, or the single-molecule real-time DNA sequencing platform PacBio (Pacific Biosciences), have recently been gaining space as a means of rapid, small scale microsatellite development (Wei *et al.*, 2014).

We used the SOLiD platform to sequence three penguin genomes and not only search for microsatellite loci, but also characterize them according to polymorphism, distribution in the genome and orthology between species, with the aim of contributing to the increase in the number of comparative studies between *Pygoscelis* species using markers with high resolution.

We observed that the number of repeats and motifs varied between the three *Pygoscelis* species. We found large amounts of dinucleotides, followed by tri- and tetranucleotides in *Pygoscelis*, as described in other vertebrate genomes (*e.g.*, Huang *et al.*, 2015). However, the relative abundance of different motif classes (*e.g.* dinucleotide) and motif types (*e.g.* AT, ACC, AAAT) is not conserved when compared with other vertebrate species. For example, Castoe *et al.* (2010) identified in the copperhead snake (*Agkistrodon contortrix*) a total of 14,612 simple sequence repeats (SSRs), 4,564 of which had flanking sequences suitable for 454 shotgun genome sequencing. These represent various amounts of different motif classes, with numbers of tetranucleotides, followed by trinucleotides and dinucleotides. The most frequent motifs were also different from those we found in our study (TC, ATT, ATCT; Castoe *et al.*, 2010). McCulloch and Stevens (2011) used Roche FLX (Titanium) Genome Sequencing to identify microsatellites (29,721 di-, tri-, tetranucleotide) for phyllostomid bats, and the authors found a majority of tetranucleotides, followed by dinucleotides and trinucleotides. Recently, 48 bird species genomes, representing all orders of Aves, were completed for phylogenetic reconstruction, which also provide a large amount of data and microsatellites to be evaluated for comparative analyses (Jarvis *et al.*, 2014).

Primers for tetranucleotides in *Pygoscelis* were designed with the same annealing temperature to enable multiplex PCR assays, which is a more affordable and time-efficient technique to genotype multiple loci and individuals. In this way, it is possible to genotype up to eight loci for each individual in a single run. Moreover, the small size (< 200 bp) of several fragments identified in the *Pygoscelis* genome allows easy amplification of low quality or low concentration DNA, such as that extracted from non-invasive (*e.g.* feather, feces) or ancient (*e.g.* museum or palaentological) samples. To this date, non-invasive DNA samples have not been used to conduct population genetics or ecological studies in penguin species due to the difficulty of obtaining good quality material for nuclear marker amplification. These samples have mostly been used for studying penguin diets, sex identification (*e.g.*, Jarman *et al.*, 2002; Deagle *et al.*, 2007; Constantini *et al.*, 2008), or studying the evolution of species using mtDNA from sub-fossil bones (*e.g.* Lambert *et al.*, 2002; Ritchie *et al.*, 2004).

In this study, we provide 34 novel polymorphic microsatellite markers, 15 of which are polymorphic in all three species and can easily be genotyped using multiplex PCR. Recently, Moodley *et al.* (2015) used NGS to develop markers for the thin-billed prion (*Pachyptila belcheri*), a sub-Antarctic seabird, and evaluated cross-amplification in six other seabird species, finding that heterozygosity decreased while proportion of non-amplifying loci increased with phylogenetic distance. In our case, similar heterozygosity was observed for all *Pygoscelis* species using loci cross-amplification, which suggests that these markers are not subject to the heterozygosity decrease among closely related species. Moreover, this was confirmed by the orthology of most loci selected for all species with same or similar primer sequences.

These polymorphic markers allow individual discrimination and their suitability for use in non-invasive or museum samples promotes their application in different types of research, such as species monitoring in the face of climate change, parentage analyses, determination of sample origin and of population genetics structure. Moreover, the same markers can be employed in comparative phylogeographical and population genetics studies of *Pygoscelis* species, given that the 15 loci developed in this study easily cross-amplify, are orthologous and widely distributed in the genome.

### Polymorphisms and utility for investigation of penguin populations

We report a large number of microsatellite loci identified in the genomes of three *Pygoscelis* species using NGS and classified according to their level of polymorphism, which may also be applied to the study of other penguin species. Previously, only a limited number of microsatellite loci were available for penguins. Twelve loci had been developed for the Humboldt penguin (Schlosser *et al.*, 2003; 2009), five for *Spheniscus* species (Akst *et al.*, 2002), and six for the Adélie penguin (Roeder *et al.*, 2001; 2002). These had been identified using traditional methods and possessed varying degrees of polymorphism in different species. In general, the same markers from these three studies have been used in population genetics investigations of several different penguin species.

Although penguins have been associated with philopatric behavior, which is often correlated with significant population structure, all studies so far have shown little or no population structure. Absence of population genetic structure was observed in Galapagos penguins using five microsatellite loci (Nims *et al.*, 2008), while Bouzat *et al.* (2009), using only four loci in samples from six colonies of Magellanic penguins, found limited genetic structure. Roeder *et al.* (2001) analyzed seven microsatellite loci in Adélie penguin samples collected from 13 localities and did not identify genetic differentiation between colonies around the Antarctic continent, with pairwise *F*_*st*_ below 0.02 and an overall *F*_*st*_ equal to 0.0007. Trakiiska *et al.* (2005) evaluated three loci in *P. papua* from Livingston Island, however, only one of them was polymorphic. Overeem *et al.* (2008) studied the population genetics of *Eudyptula minor* using five loci in samples from seven colonies and found that most were genetically homogeneous. Likewise, Schlosser *et al.* (2009) found reduced genetic structure in the Humboldt penguin along its distribution based on 12 microsatellite loci. Sakaoka *et al.* (2014) used eight microsatellite loci to understand the breeding behavior of *P. adeliae*, revealing an absence of extra-pair paternity (EPP) in a particular captive population. All the aforementioned studies used an average of 6 loci for penguin population genetics studies, most of which using the same or similar marker combination. If these markers do not provide enough resolution to reflect species behavior and ecology, then doubt is cast on the studies’ findings. Recently, Vianna *et al.* (2017), used a total of 12 loci selected from this study (Table 4) to understand population genetics structure of *P. papua*. Significant population structure was identified between *P. papua* colonies from sub-Antarctic (Falkland/Malvinas, Crozet and Kerguelen Islands) and those from WAP, but also among nine localities along WAP. These results put in evidence the resolution of our selected markers to detect population genetics structure in this species. Other loci developed for penguins were often not evaluated in a population study or showed population structure. Ahmed *et al.* (2009) developed 25 loci for the Macaroni penguin, eight to 12 loci were found to be polymorphic, and Kang *et al.* (2015) isolated 16 polymorphic microsatellite loci for the Chinstrap penguin (*P. antarcticus*). However, those markers were not employed in population genetics investigations. Boessenkool *et al.* (2008, 2009) developed 12 other microsatellite loci for the Yellow-eyed penguin (*Megadyptes antipodes*), and found two distinct genetic groups based on that dataset.

This elicits different questions: are penguins really philopatric, highly migrant, or both? Are species with such large geographical distributions, living under different environmental conditions and subject to various barriers, really that genetically homogeneous? Or should more highly variable markers be used to obtain the appropriate resolution for the type of questions asked? For several kinds of population genetics data analyses, results are sensitive to the number of loci used and their polymorphic information content, as well as the number of populations sampled and of individuals typed in each population. Evanno *et al.* (2005), for instance, suggest that a minimum of 10 polymorphic microsatellites are required to detect population genetic structure using the Bayesian algorithm implemented in Structure software.

There is an increasing amount of research using SNPs with genomic methods, such as GBS, RADseq or ddRAD for population studies, including recent studies in penguins (*e.g.* Clucas *et al.*, 2016; Cristofari *et al.*, 2016). However, microsatellites will remain useful for population studies when discrepant patterns are observed between markers (mtDNA, microsatellite, SNPs). This is the case of three clades identified for the emperor penguin using mtDNA (Younger *et al.*, 2015) and the lack of population structure using genome-wide SNPs (Cristofari *et al.*, 2016). Apart of population studies, microsatellites are very useful for studies such as individual-based identification or paternity assessment. Therefore, it is important to identify polymorphic microsatellite loci in non-model species, especially those inhabiting areas highly impacted by climate change, such as the Antarctic. We identified about 11,849 tetranucleotide markers and selected 34 which were polymorphic, cross-amplified in all three *Pygoscelis* species, and did not show signs of heterozygosity reduction due to cross-amplification or due to orthology identified in the majority of those loci selected. We believe that the microsatellite markers developed in this study will help to gain a comprehensive understanding of *Pygoscelis* penguin population genetics and ecology.

## Supplementary Material

The following online material is available for this article:

Figure S1 - Probability of identity (PI), and the PI between siblings (PI_sib_) for the 15 microsatellite loci.

Table S1 - All tetranucleotide loci for *P. adeliae, P. papua P. antarcticus*.
