# Supplementary material for: Comparative genome-wide polymorphic microsatellite markers in Antarctic penguins through next generation sequencing
Source: Genet Mol Biol. 2017 Jul-Sep;40(3):676–87. doi: 10.1590/1678-4685-GMB-2016-0224 (PMC5596379; doi:10.1590/1678-4685-GMB-2016-0224)
Supplement: Supplementary file 2 [file 1415-4757-gmb-40-03-0676-Suppl01.pdf]

Genetics and Molecular Biology, 40, 3 (2017)

Supplementary Material to "Comparative genome-wide polymorphic microsatellite markers in Antarctic penguins through next generation sequencing"

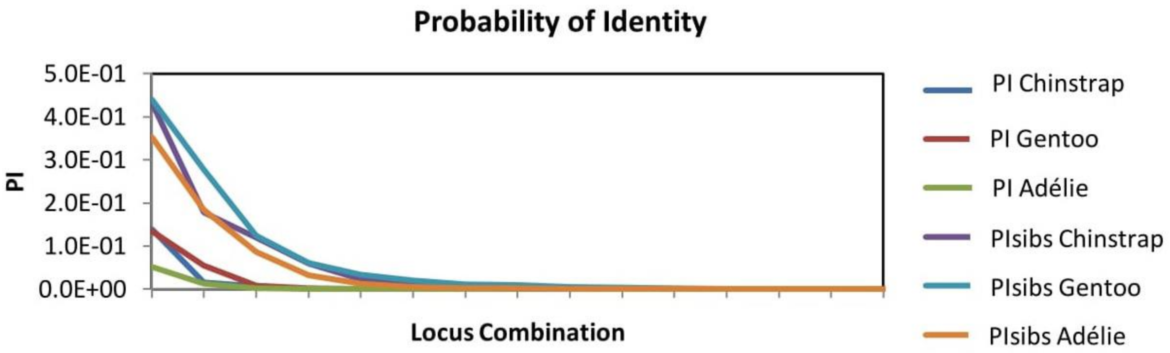

Figure S1 – Probability of identity (PI), and the PI between siblings (PI_{sib}) for the 15 microsatellite loci, which is the number of loci required to resolve individual identity within populations by gradually increasing the number of loci (locus combination), adding the most variable loci first.
